# Quantum speed limits in open systems: Non-Markovian dynamics without rotating-wave approximation

**DOI:** 10.1038/srep08444

**Published:** 2015-02-13

**Authors:** Zhe Sun, Jing Liu, Jian Ma, Xiaoguang Wang

**Affiliations:** 1 Department of Physics, Hangzhou Normal University, Hangzhou 310036, China; 2 Department of Physics, National University of Singapore, Singapore 117542; 3 Singapore University of Technology and Design, 20 Dover Drive 138682, Singapore; 4 Zhejiang Institute of Modern Physics, Department of Physics, Zhejiang University, Hangzhou 310027, China

## Abstract

We derive an easily computable quantum speed limit (QSL) time bound for open systems whose initial states can be chosen as either pure or mixed states. Moreover, this QSL time is applicable to either Markovian or non-Markovian dynamics. By using of a hierarchy equation method, we numerically study the QSL time bound in a qubit system interacting with a single broadened cavity mode without rotating-wave, Born and Markovian approximation. By comparing with rotating-wave approximation (RWA) results, we show that the counter-rotating terms are helpful to increase evolution speed. The problem of non-Markovianity is also considered. We find that for non-RWA cases, increasing system-bath coupling can not always enhance the non-Markovianity, which is qualitatively different from the results with RWA. When considering the relation between QSL and non-Markovianity, we find that for small broadening widths of the cavity mode, non-Markovianity can increase the evolution speed in either RWA or non-RWA cases, while, for larger broadening widths, it is not true for non-RWA cases.

The problem of how fast can a quantum system evolve is of particular interest and has attracted much attention. Quantum mechanics, as a fundamental law of nature, provides ultimate constraints known as quantum speed limits (QSLs) which are virtually at the center of all areas of quantum physics and thus they are of manifold applications, including exploring the physical limits of computation^1^, providing fundamental limit of precision under quantum metrology^2^,^3^, restricting efficiency of quantum optimal control algorithms^4^,^5^ and providing a minimal time bound to perform the optimal process^6^. The maximal rate *υ* of evolution can be described by the QSL time defined as the minimal time *τ*_QSL_ needed to evolve the initial state (pure or mixed) *ρ*_0_ to a target state *ρ_t_* through a unitary evolution acted by a time-independent Hamiltonian *H*, i.e., the shorter time *τ*_QSL_ means the higher rate *υ*. The evolution time is lower-bounded by Refs. 7,8,9:
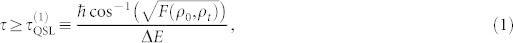
where 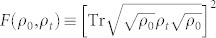 is the Bures fidelity which can be used to characterize the distance between the two states, and 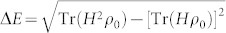 is the standard deviation of the initial-state energy. On the other hand, some researchers found that in several cases, the evolution time can be bounded more tightly by the average energy above the ground state, *E* − *E*_0_^10^,^11^, namely,
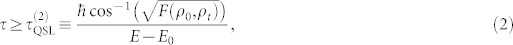
where the initial state *ρ*_0_ and target state *ρ_t_* can be pure or mixed, and the energies of the initial state is *E* = Tr(*Hρ*_0_) and the ground state energy is *E*_0_ = Tr(*Hρ*_ground_). Therefore, based on the two results above, people usually define the QSL time as 
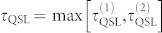
.

Most previous studies focus on unitary dynamics of isolated quantum systems^7^,^8^,^9^,^10^,^11^,^12^,^13^,^14^,^15^, while, all systems are unavoidably coupled to their environments. Therefore, it is necessary to determine a QSL time for open systems^16^,^17^,^18^,^19^. Recently, Taddei *et al*.^16^ developed a method to investigate the QSL problem in open systems described by positive non-unitary maps by using of quantum Fisher information for time estimation. While, for the case of the initial mixed states, a Hermitian operator is required to minimize the Fisher information in the enlarged system-environment space, which is generally a challenging task. Soon later, del Campo *et al*.^17^ employed the concept of *relative purity* to derive an analytical and computable QSL time for open systems undergoing a completely positive and trace preserving evolution. Actually, their bound also easily accounts for the non-Markovian dynamics. It should be noted that *relative entropy* can perfectly make a distinction between an initial pure state and its target state, however it may fail to distinguish an initial mixed state to its target state. Because in the latter case, relative purity can reach the value of 1, even though the two states are not completely consistent (see examples in the following section). Recently, Deffner and Lutz^18^ formulated a tight bound on the minimal evolution time of an arbitrarily driven open system, and showed that non-Markovian effects can speed up quantum evolution. However, their time bound is derived from pure initial states and can not be directly applied into the mixed initial states.

Therefore, motivated by the recent studies above, we employ an alternative fidelity definition different from *relative entropy* in order to derive a new computable QSL time bound, which can easily account for the situations where the initial states are mixed. From this point of view, we emphasize that the new QSL time bound is superior to all the previous bounds in the cases of initial mixed states. On the other hand, the system-environment interaction will introduce quantum decoherence, which is one of the most important problem in quantum information processing^20^. How does the decoherence process affect the QSL time, consequently, affect the evolution speed of the system? In order to give a constructive answer, we study the QSL time problem in a qubit system interacting with a bosonic bath. Moreover, by using of a hierarchy equation method, we try to find the effects of counter-rotating terms and non-Markovianity on the QSL time bound, which are short of study in the previous literatures.

Usually, the description of the dynamics of open systems involves various approximations, such as the Born and Markovian approximation. An effective method that avoids the above two approximations was developed by Tanimura *et al*.^21^,^22^,^23^,^24^,^25^, who established a set of hierarchical equations that includes all orders of system-bath interactions. The derivation of the hierarchy equations requires that the time-correlation function of the bath can be decomposed into a set of exponential functions^25^. At finite temperature, this requirement is fulfilled if the system-bath coupling can be described by a Drude spectrum. The hierarchy equation method has been successfully used in describing quantum dynamics of chemical and biophysical systems^23^,^24^,^25^,^26^,^27^,^28^. On the other hand, the hierarchy equation method is also powerful to study the dynamics of qubit devices at low operating temperature^29^, when the environment is usually modeled by a Lorentz-broadened cavity mode.

The set of hierarchy equations derived here provides an exact treatment of decoherence, and employs neither the rotating-wave, Born, nor Markovian approximations. The hierarchy equation method enables us to deeply explore the effects of the environment on the QSL time bound, which is presented as follows: (i) system-bath correlations are here fully accounted during the entire time evolution, which is different from that the correlations are truncated to second order. High-order correlations are shown^30^ to be very important, even producing a totally different physics; (ii) without weak coupling approximation, the hierarchy equation method is a promising method for studying strong- and ultrastrong- coupling physics^31^,^32^. (iii) Refs. 33 and 34 found that the RWA may lead to faulty results. Especially, recent developments in physical implementation lead to strong coupling between qubit and cavity modes^31^,^32^, which requires a careful consideration of the effect of counter-rotating terms. Fortunately, the RWA can be avoided in hierarchy equation method; (iv) Markovian approximation is naturally avoided in hierarchy equation method, thus we can consider the effects of non-Markovianity. Recently more and more attention and interest have been devoted to the study of non-Markovian processes^35^,^36^,^37^,^38^,^39^,^40^,^41^,^42^.

In the following sections, we will first give the QSL time bound derived from a fidelity which is different from the *relative purity* and Bures fidelity. Secondly, we synoptically introduce hierarchy equation method, then we will consider the QSL time in a qubit system interacting with a broadened cavity mode. The qubit-cavity coupling spectrum is described as Lorentz type. The results obtained by hierarchy equations will be compared with those obtained within RWA. The relation between QSL and non-Markovianity will also be explored.

## Results

### Derivation of quantum speed limit time

Firstly, we should employ the concept of fidelity as the distance measure of two quantum states. It is well known that the Bures fidelity may be a perfect definition of fidelity^43^. However, due to the difficulty in the calculation, people try to find alternative definitions of fidelity^44^,^45^,^46^. Among them, we find the definition studied by Wang *et al*. from the point of Hilbert–Schmidt product for two operators^46^, has some desirable properties and could be a good distance measure for two states density. The definition reads
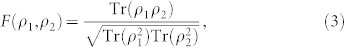
where the *ρ*_1_, *ρ*_2_ denote two arbitrary density matrices This fidelity *F* satisfies Jozsa's four axioms^43^ up to a normalization factor that:

1. *F* is normalized. The maximum 1 is attained if and only if *ρ*_1_ = *ρ*_2_;
2. *F* is invariant under swapping the two states, i.e., *F* (*ρ*_1_, *ρ*_2_) = *F* (*ρ*_2_, *ρ*_1_);
3. The fidelity is invariant under unitary transformation *U* on the state space, i.e., *F* (*Uρ*_1_*U*, *Uρ*_2_*U*) = *F* (*ρ*_1_, *ρ*_2_);
4. When one of the state is pure, say, *ρ*_2_ = |*ψ*〉 〈*ψ*|, the fidelity reduces to 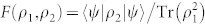.

In addition to these advantages, the fidelity (3) is relatively easy to calculate since it only contains the Hilbert–Schmidt inner product and purity. Therefore, it has been well applied in experiment studies such as in NMR system^47^. Here, it should be compared with the definition of *relative purity* used in Ref. 17, i.e., 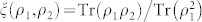. One can easily find that the *relative purity ξ* fails to satisfy axioms (1) and (2) for general mixed states, e.g., for two different states 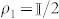 (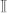 is 2-dimensional identity matrix) and *ρ*_2_ an arbitrary 2-dimensional density matrix, one can still obtain *ξ* (*ρ*_1_, *ρ*_2_) = 1. From this point of view, *relative purity* is not suitable to act as a distance measure when the initial state of *ρ*_1_ is a mixed state, and thus it may induce some defects into the derivation of QSL time for this case.

Let us now calculate the changing rate of the fidelity (3). By denoting the initial state as *ρ*_0_ and the state at time *t* as *ρ_t_*, the derivative of fidelity *F* (*ρ*_0_, *ρ_t_*) becomes
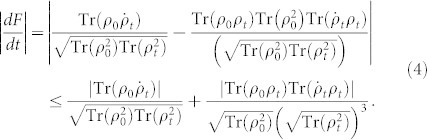
The rate above can be further bounded by using Cauchy-Schwarz inequality for operators, i.e., |Tr (*AB*)|^2^ ≤ Tr(*AA*)Tr(*BB*), then we have
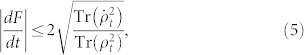
Integrating Eq. (5) over a time period *τ* leads to the following inequality
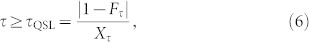
where *F_τ_* = *F* (*ρ*_0_, *ρ_τ_*) denotes the target value of the fidelity in Eq. (3) at time *τ*, and the kernel parameter is defined as
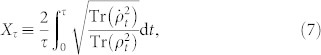
where we retain the time derivative 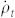 in order that the time limit *τ*_QSL_ in Eq. (6) can be used to consider either Markovian or non-Markovian dynamics.

We should note that for the case of a initial pure state |*ψ* (0)〉 under a unitary evolution 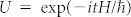, then we have 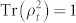, consequently, 
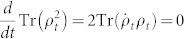
. Thus the second term in the first line in Eq. (4) equals zero and the coefficient 2 in *X_τ_* should be omitted. Furthermore, the minimum time required for the time evolving state |*ψ* (*t*)〉 to become orthogonal to its initial state |*ψ* (0)〉, i.e., the so-called passage time^12^, 
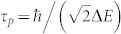
, with (Δ*E*)^2^ = 〈*ψ* (0)| *H*^2^ |*ψ* (0)〉 − 〈*ψ* (0)| *H* |*ψ* (0)〉^2^, which is consistent with that of Ref. 17. However, for mixed initial states and non-unitary evolutions, our result will be inevitably different from that of Ref. 17.

Moreover, when we take into account a fact that 
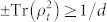
 (with *d* the dimension of *ρ_t_*), then we will obtain a looser time bound by substituting 
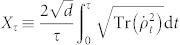
 into Eq. (6). Consequently, when considering a Lindblad-form evolution, we will provide a QSL time bound depending on the initial state and the generators of the dynamical channel similar to the result in Ref. 17.

### The system-environment model and the hierarchy equation method

Here we consider qubits interacting with a bosonic bath, also known as the spin-boson model:
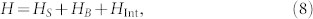
where *H_S_* is the free Hamiltonian of the qubit (with 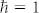), and here we choose
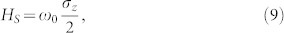
where *σ_z_*_(*x*,*y*)_ is the Pauli operator, and
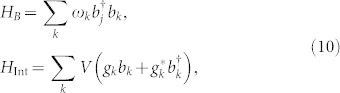
where *V* is the operator of the qubit and here we choose *V* = *σ_x_*. 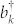 and *b_k_* are the creation and annihilation operators of the bath, while *g_k_* is the coupling strength between the qubit and the *k*th mode of the bath.

The exact dynamics of the system in the interaction picture can be derived as Ref. 25
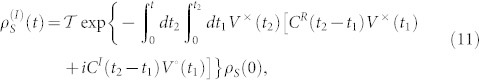
if the qubit and bath are initially in a separable state, i.e. 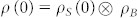, where *ρ_B_* is the initial state of the bath. In Eq. (11), 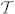 is the chronological time-ordering operator, which orders the operators inside the integral such that the time arguments increase from right to left. Two superoperators are introduced, *A* ^×^ *B* ≡ [*A*, *B*] = *AB* − *BA* and *A*°*B* ≡ {*A*, *B*} = *AB* + *BA*. Also, *C^R^* (*t*_2_ − *t*_1_) and *C^I^* (*t*_2_ − *t*_1_) are the real and imaginary parts of the bath time-correlation function
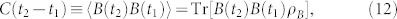
respectively, and
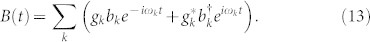
Equation (11) is difficult to solve directly, due to the time-ordered integral. An effective method for this problem was developed^21^,^22^,^23^,^24^,^25^,^26^,^27^ by solving a set of hierarchy equations, such as the form of Eq. (17). A key condition in deriving the hierarchy equation is that the correlation function (13) should be decomposed into a sum of exponential functions of time as 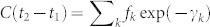, with parameters *f_k_* and *γ_k_* depend on the system-bath coupling spectrum and the temperature. Then, the hierarchy equations are obtained by repeatedly taking the derivative of the right-hand side.

At finite temperatures, the system-bath coupling can be described by the Drude spectrum, however, when we consider qubit devices, which are generally prepared in nearly zero-temperature environments. Then the coupling spectrum between the qubits and cavity modes is usually Lorentz type, and the hierarchy method can also be applied^29^.

Now we consider one qubit interacting with a single mode of the cavity, with transition frequency *ω*_0_. Due to the imperfection of the cavity, the single mode is broadened and the qubit-cavity coupling spectrum becomes Lorentz-type
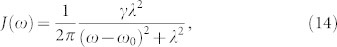
where *λ* is the broadening width of the cavity mode which is connected to the bath correlation time 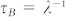. The relaxation time scale on which the state of the system changes is related to *γ* by *τ_s_* = *γ*^−1^, and *γ* partly reflects the system-bath coupling strength, because when integrating the spectrum *J* (*ω*) over the entire region of *ω*, one will give the effective coupling strength as 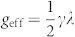.

At zero temperature, if the cavity is initially in a vacuum state 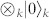, the time-correlation function (13) becomes
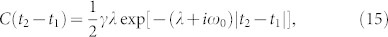
which is an exponential form that we need to use for the hierarchy equations. For a limit case, 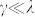, i.e., 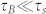, then we have a flat spectrum of Eq. (14) and the correlation tends to *δ* function that *C* (*t*_2_ − *t*_1_) → *γδ* (*t*_2_ − *t*_1_), this is the so-called Markovian limit and the Markovian decay rate *γ_M_* = *γ*.

To derive the hierarchy equation in a convenient form, we further write the real and imaginary parts of the time-correlation function (15) as
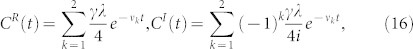
where *ν_k_* = *λ* + (−1)*^k^iω*_0_. Then, following procedures shown in Refs. 21, 25, the hierarchy equations of the qubits are obtained as
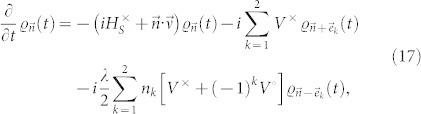
where the subscript 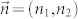 is a two-dimensional index, with integer numbers *n*_1(2)_ ≥ 0, and 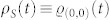. The vectors, 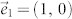, 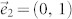, and 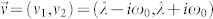. We emphasize that 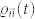 with 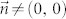 are auxiliary operators introduced only for the sake of computing, they are not density matrices, and are all set to be zero at *t* = 0. The hierarchy equations are a set of linear differential equations, and can be solved by using the Runge-Kutta method. The contributions of the bath to the dynamics of the system, including both dissipation and Lamb shift, are fully contained in the hierarchy equation (17). The Lamb shift term, which is related to the imaginary part of the bath correlation function, can be written explicitly in the common non-Markovian equations. Since the real and imaginary parts of the bath correlation function are taken into considered here, the effects of the Lamb shift exist in the hierarchy equations, although not in an explicit form.

For numerical computations, the hierarchy equation (17) must be truncated for large enough 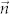. We can increase the hierarchy order 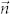 until the results of *ρ_S_*(*t*) converge. The terminator of the hierarchy equation is
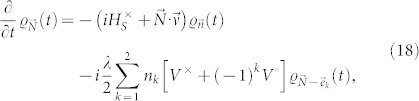
where we dropped the deeper auxiliary operators 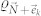. The numerical results in this paper were all tested and converged, and the density matrix *ρ_S_*(*t*) is positive. The detailed derivation of Eq. (17) can be found in Refs. 21, 29.

### Numerical results

In Fig. 1, for a pure initial state, i.e., 
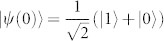
, where |1〉, |0〉 denote the eigenstates of the Pauli operator *σ_z_*, we plot *τ*_QSL_ of Eq. (6) versus parameter *γ* by using hierarchy equation method (*γ* and *λ* are in units of *ω*_0_, which is omitted in the following for simplicity). The system-bath interaction Hamiltonian is 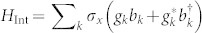, so non-RWA case is considered here. The broadening-width parameter *λ* = 0.2 and the actual evolution time is *t* = 30. For comparison, we also plot 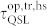 which are derived from operator norm, trace norm and Hilbert-Schmidt norm respectively in Ref. 18. For a Hermitian operator *A*, if its singular values are *μ_i_*, the operator norm is given by the largest singular value 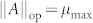, the trace norm is equal to the sum 
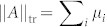
, and the Hilbert-Schmidt norm is defined as 
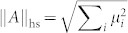
. Subsequently, the QSL time 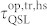 can be obtained by substituting 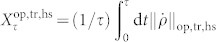 into Eq. (6). From numerical results, one can find that 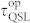 corresponding to the highest curve is the tightest bound. And our QSL time *τ*_QSL_ presents the lowest values in most region of *γ*, except for small values of *γ* it gives tighter bound than 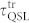. Despite the fact that our bound is not tight, it presents similar behavior with the tighter bounds. In the sense of the application in mixed state cases, it allows us to explore the effects of the parameters of the driven Hamiltonian and the purity of the initial states on the QSL time in later parts.

In the following figures, we choose a mixed initial state of Werner-type:
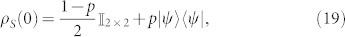
where 
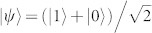
, the parameter 0 ≤ *p* ≤ 1, and 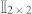 denotes a 2 × 2 identity matrix.

In Fig. 2, Fig. 3, and Fig. 4, we numerically investigate QSL time bound *τ*_QSL_ versus parameter *γ*. A reasonable comparison between the solutions within and without RWA will enable us to understand the contribution of the counter-rotating terms. The analytical results of the density matrix at arbitrary time *t* for RWA case is shown in the *method* section. Moreover, in order to find the relation between QSL time and non-Markovianity, we also numerically plot the measure of non-Markovianity *M* for RWA and non-RWA cases. Physically speaking, non-Markovian dynamics implies that the distinguishability of the pair of states increases at certain times. This can be interpreted as a flow of information from the environment back to the system, which prevents the coherence information loss of the system and thus helps to distinguish the two states. Therefore, it is nature to consider that whether non-Markovianity can accelerate the evolution of the system. The definition of the measure *M* is shown in the *method* section.

In Fig. 2a, a small broadening width of the cavity mode *λ* = 0.03 is chosen, then increasing parameter *γ* means increasing the qubit-cavity coupling strength. We find that for a long region of *γ* from 0 to about 26.46, *τ*_QSL_ of non-RWA case (dotted black line) is not longer than that of RWA case (blue line with circles), which means the counter-rotating-wave term retained in the non-RWA case can reduce the *τ*_QSL_, i.e., increase the evolving speed. While, when *γ* is too large, *τ*_QSL_ of non-RWA increases quickly and becomes larger than that of RWA case.

Fig. 2b shows the non-Markovianity measure *M* versus parameter *γ*. In RWA case (blue line with circles), *M* increases with increasing *γ*, while in non-RWA case (dotted black line), it is obviously different. Due to the counter-rotating terms, it is no longer the case that larger *γ* can induce greater non-Markovianity. When *γ* is larger than a certain value, *M* begins to decline.

If contrasting the two subfigures Fig. 2a and Fig. 2b, we find that increasing non-Markovianity will decrease the *τ*_QSL_ in both RWA and non-RWA cases. While, in non-RWA case, the remarkable reducing process of *M* for larger *γ* corresponds to the increase of *τ*_QSL_. Therefore, in this small *λ* case, non-Markovianity directly affects the QSL time bound, i.e., larger non-Markovianity decreases *τ*_QSL_, while, smaller non-Markovianity will increase *τ*_QSL_.

When we choose a larger parameter *λ* = 0.1 in Fig. 3, one can see some similar behaviors of *τ*_QSL_ and non-Markovianity with Fig. 2, e.g., there is also a crossing of *τ*_QSL_ for RWA and non-RWA cases, while the crossing point occurs at a smaller value of *γ* where the non-Markovianity of non-RWA case begins to decrease.

Different phenomena are shown in Fig. 4, when we continue to enlarge the width to *λ* = 0.6, there no longer exists the crossing of *τ*_QSL_ in the two cases of RWA and non-RWA. Instead, *τ*_QSL_ of non-RWA is always lower than that of RWA case in the presented region of *γ*. Different dependence behaviors of *τ*_QSL_ on *M* are also found for the non-RWA case. When the non-Markovianity decreases (dotted black line in Fig. 4b), *τ*_QSL_ (dotted black line in Fig. 4a) also decreases, which is quite different from the former results in Fig. 2 and Fig. 3 where *λ* is smaller.

Therefore, if someone draws the conclusions that non-Markovianity can decrease QSL time *τ*_QSL_^18^, our results will provide important additions and amendments that with using RWA, non-Markovianity can indeed decrease QSL time *τ*_QSL_. However, without using RWA, only in the condition of small broadening-width parameter *λ*, increasing non-Markovianity can depress *τ*_QSL_. On the contrary, decreasing non-Markovianity will increase *τ*_QSL_. However, if the width *λ* is large enough, the dependence of *τ*_QSL_ on non-Markovianity will change. Instead, non-Markovianity reaches the maximum followed by that *τ*_QSL_ also gets its maximum.

On the other hand, from Fig. 2, Fig. 3 and Fig. 4, one can see the effects of the broadening-width parameter *λ*. If averaging *τ*_QSL_ over the presented region of *γ*, one can see that the QSL time in Fig. 4a is larger than that in Fig. 2a and Fig. 3a. Correspondingly, the averaged value of non-Markovianity *M* in Fig. 4b is obviously smaller than those of Fig. 2b and Fig. 3b, which implies that increasing the cavity mode width, i.e., enhancing the damping rate of the mode, will reduce the non-Markovianity and enlarge the *τ*_QSL_ and thus decelerate the evolution. We should note that the difference between RWA and non-RWA cases demonstrated by *τ*_QSL_ and *M* becomes more and more evident when the two parameters *γ* and *λ* increase. It is to say, when the time scales of the system and the bath become small, the counter-rotating terms will play an important role. Then, the double excitations followed by the virtual exchanges of energy, which are introduced by the counter-rotating terms, may prevent the information backow from the environment and thus weaken the non-Markovianity.

In Fig. 5, we plot *τ*_QSL_ versus *γ* for different initial states of Eq. (19) with different mixed coefficients *p*. Since the purity is defined as 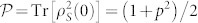. Thus, larger value of *p* corresponds to higher purity, namely closer to a pure state. Surprisingly, the non-RWA case in Fig. 5a shows some crossings of *τ*_QSL_, namely, it is not always a truth that pure state may induce lower QSL time bound than mixed states. Obviously, for larger values of *γ*, mixed state such as *p* = 0.1 which is far from pure state, can bring much lower QSL time. But for RWA case, there will not be the strange phenomena any more.

## Discussion

We have derived a computable QSL time bound which can be easily applied in the open systems of mixed initial states undergoing non-Markovian dynamics. By making use of the hierarchy equation method, we considered a qubit system coupled to a broadened cavity mode. We have found that the counter-rotating terms (in non-RWA case) can be helpful to decrease QSL times, i.e., accelerate quantum evolution. In non-RWA case, for narrow broadening-width *λ* of the cavity mode, properly enlarging the qubit-cavity coupling can decrease QSL time, however, too strong coupling will cause a quickly increasing process of QSL time. While, for wider *λ*s, there exists a maximal QSL time, after that, QSL time decreases monotonically with increasing qubit-cavity coupling strength.

On the other hand, in non-RWA case, non-Markovianity exhibits quite different behavior from RWA case. Too strong qubit-cavity coupling may weaken non-Markovianity. Our results have also demonstrated the close relationship between QSL time and non-Markovianity. Especially for narrow broadening-widths of the cavity mode, increasing non-Markovianity helps to shorten QSL time (for both RWA and non-RWA), while weaker non-Markovianity may increase QSL time (for non-RWA). While, enlarging broadening-width *λ* will weaken non-Markovianity and increase QSL time. We also considered initial states with different purity and found that in non-RWA case, the mixed state with lower purity can also lead to shorter QSL time when the qubit-cavity coupling is strong enough.

Several tighter bounds depend on exact time-evolution of the density matrix. However, if so, people prefer to consider the exact dynamics behaviour of the density rather than the bound. Therefore, the purpose of deriving a bound is to describe the evolution speed even though we have not grasped enough information of the dynamics. Moreover, the simpler the computation of the bound is, the better it will be applied. From this point of view, we found the only practical QSL bound derived for open systems is that of Ref. 17, which only depends on the initial state and the generators in the Lindblad-form evolution. Beyond that, our bound is more available in the case of initial mixed states. Certainly, it is still attractive to explore tight and practical QSL bound in open systems, and which leaves lots of interesting problems.

## Method

### Density matrix within RWA

If we make use RWA, the interaction Hamiltonian in the total Hamiltonian (8) becomes
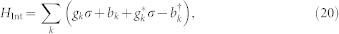
then with the Lorentz-type coupling spectrum and the vacuum initial state of the cavity mode, the density matrix for arbitrary time *t* can be obtained analytically^20^ as
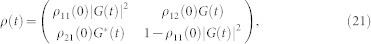
where the time-dependent parameter
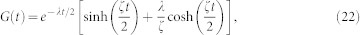
with 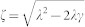.

**Measure of non-Markovianity**: Let us give a brief introduction of the measure of non-Markovianity defined by Breuer *et al*.^35^. For a quantum process, the measure is defined as:
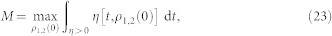
where *η* [*t*, *ρ*_1,2_ (0)] denotes the changing rate of the trace distance that
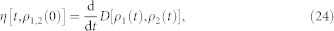
where 
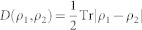
 is the trace distance of the quantum states *ρ*_1_ and *ρ*_2_ with the trace norm definition for a operator 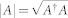. The distance *D* above characterizes the distinguishability between two quantum states and satisfies 0 ≤ *D* ≤ 1. It has been pointed that all completely positive and trace-preserving (CPT) maps cannot increase the distance *D*. For example, *η* ≤ 0 for all dynamical semigroups and all time-dependent Markovian processes, while, if there exists a pair of initial states and a certain time interval such that *η* > 0, then we can say that the non-Markovianity appears.

It should be noted that the time integration in Eq. (23) is extended over all time intervals (*a_i_*, *b_i_*) in which *η* is positive, and the maximum is taken over all pairs of initial states. The measure can be rewritten as:
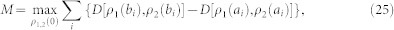


## Figures and Tables

**Figure 1:**
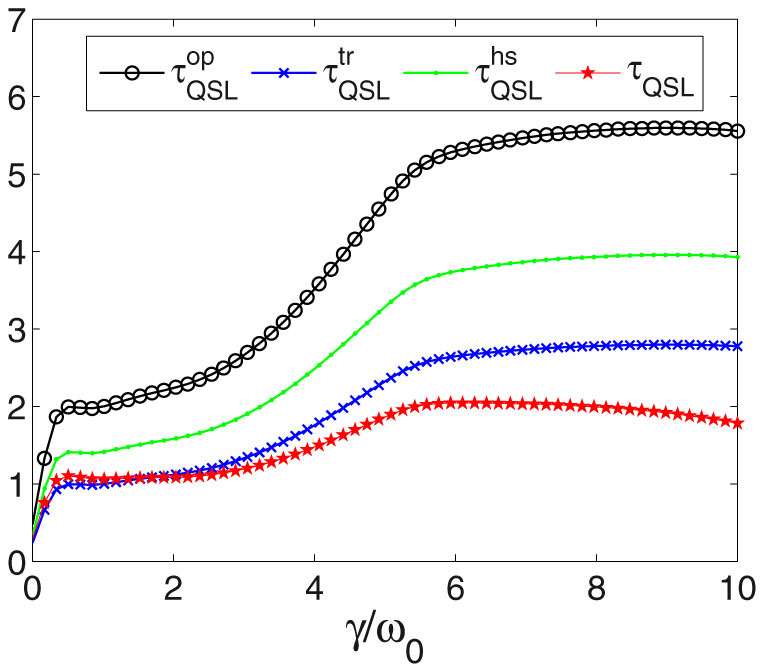
Quantum speed limit time versus different parameter *γ* (in units of *ω*_0_). The initial state is pure state. Different QSL time definitions are shown. 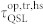 are derived from the operator norm, trace norm and Hilbert-Schmidt norm in Ref. 18. The actual driving time *τ* = 30.

**Figure 2:**
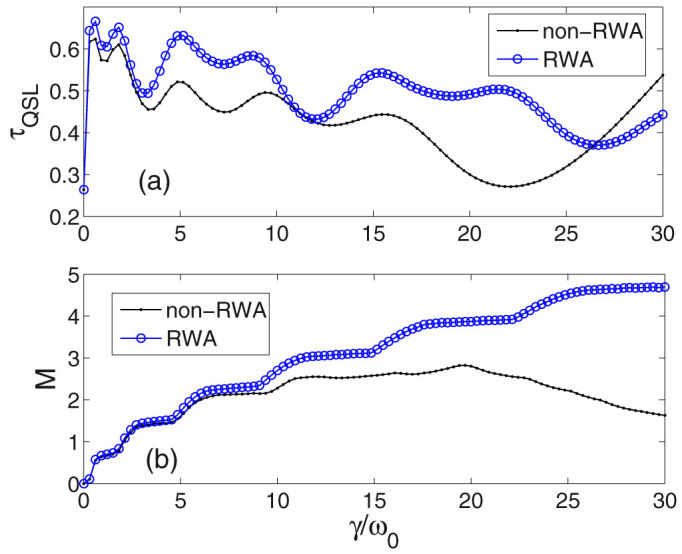
(a) Quantum speed limit time versus different parameter *γ* (in units of *ω*_0_). The broadening-width parameter *λ* = 0.03 (in units of *ω*_0_). The initial state is chosen as a mixed state in Eq. (19) with mixed parameter *p* = 0.8. (b) Measure of non-Markovianity *M* versus different parameter *γ*. The cases of non-RWA (black solid line with dots) and RWA (blue solid line with circles) are plotted. The actual driving time *τ* = 30.

**Figure 3:**
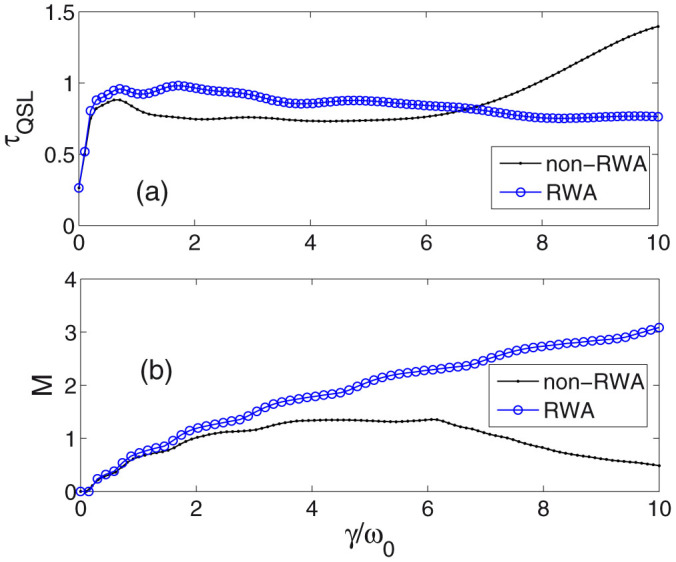
(a) Quantum speed limit time versus different parameter *γ* (in units of *ω*_0_). The broadening-width parameter *λ* = 0.1 (in units of *ω*_0_). (b) Measure of non-Markovianity versus different parameter *γ*. The cases of non-RWA (black solid line with dots) and RWA (blue solid line with circles) are plotted. The initial state and other parameters are chosen as Fig. 2.

**Figure 4:**
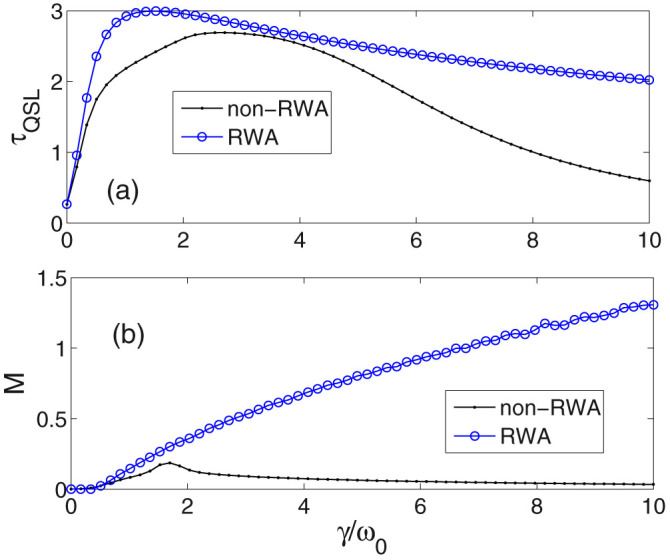
(a) Quantum speed limit time versus different parameter *γ* (in units of *ω*_0_). The broadening-width parameter *λ* = 0.6 (in units of *ω*_0_). (b) Measure of non-Markovianity versus different parameter *γ*. The cases of non-RWA (black solid line with dots) and RWA (blue solid line with circles) are plotted. The initial state and other parameters are chosen as Fig. 2.

**Figure 5:**
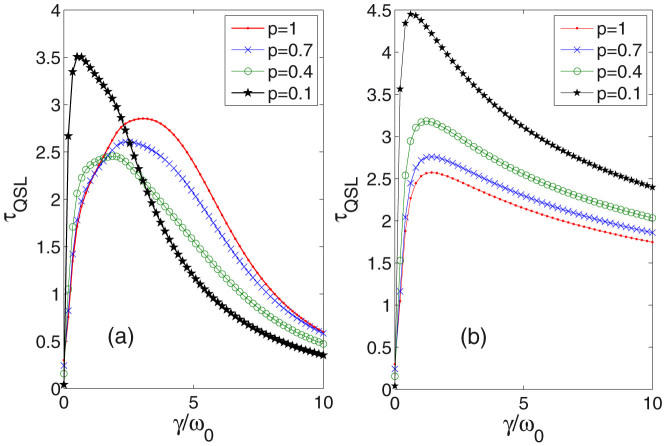
(a) For the non-RWA case, we plot quantum speed limit time versus different parameter *γ* (in units of *ω*_0_). The broadening-width parameter *λ* = 0.6 (in units of *ω*_0_). Different initial states are studied by choosing different parameters *p* = 1, 0.7, 0.4, 0.1. For comparison, we plot the RWA case in (b).
